# Synthesis, Colloidal Characterization and Targetability of Phenylboronic Acid Functionalized α-Tocopheryl Polyethylene Glycol Succinate in Cancer Cells

**DOI:** 10.3390/polym12102258

**Published:** 2020-10-01

**Authors:** Sanjay Tiwari, Jayant Sarolia, Vrushti Kansara, Nishith A. Chudasama, Kamalesh Prasad, Debes Ray, Vinod K Aswal, Pratap Bahadur

**Affiliations:** 1 Maliba Pharmacy College, Gopal-Vidyanagar Campus, Uka Tarsadia University, Surat 394350, India; sanjay.tiwari@utu.ac.in (S.T.); jayantsarolia@gmail.com (J.S.); v.kansara12@gmail.com (V.K.); 2 Natural Products & Green Chemistry Division, Central Salt and Marine Chemicals Research Institute, Bhavnagar 364002, India; chudasamnishith@gmail.com (N.A.C.); kamlesh@csmcri.res.in (K.P.); 3 Solid State Physics Division, Bhabha Atomic Research Centre, Mumbai 400085, India; debes@barc.gov.in (D.R.); vkaswal@barc.gov.in (V.K.A.); 4 Department of Chemistry, Veer Narmad South Gujarat University, Surat 395007, India

**Keywords:** micelles, TPGS, functionalization, drug targeting, phenylboronic acid, paclitaxel

## Abstract

This study reports targetable micelles developed after covalent functionalization of α-tocopheryl polyethylene glycol succinate (TPGS) with amino phenylboronic acid (APBA). Nuclear magnetic resonance (NMR) and infrared (IR) spectroscopic results showed successful attachment of APBA to the hydrophilic segment of TPGS. Dynamic light scattering and small-angle neutron scattering studies revealed that the conjugate self-assembled in water to produce spherical core-shell micelles (14–20 nm) which remained stable against temperature (ca. 25–45 °C) and pH changes. The micelles could solubilize a high payload of paclitaxel (PLX) without exhibiting changes in the average size. However, at the saturation solubility, drug molecules migrated from the core to the shell region and engaged with APBA groups via π–π stacking interaction. Confocal microscopy and cell sorting analyses verified the effective translocation ability of TPGS-APBA micelles in sialic acid (SA) expressing MDA-MB-453 cells. At equivalent PLX dose, TPGS-APBA micelles showed about a twofold improvement in apoptotic death among the cells exposed for 2 h. Our findings indicate that the attachment of APBA can be a potential strategy for improving the intra-cellular localization of carriers among cancer cells expressing SA residues.

## 1. Introduction

The terminal position of cell membrane glycoproteins and glycolipids is occupied by N-acetylneuraminic acid derivatives. The latter is dominated by sialic acid (SA), a nine carbon sugar which imparts a net negative charge to cells [1,2]. Cancer progression correlates with the addition and branching of terminal SA groups (hypersialylation) over the cell surface. Triggered through upregulated action of cytosolic sialyltransferases (ST), hypersialylation configures the microenvironments conducive to cancer cell progression, metastasis and immune evasion [3,4]. Therapeutic strategies aimed at blocking SA synthesis (ST inhibitors) or cleavage of SA branching with neuraminidase have been criticized for poor transcellular permeation and low efficacy [5,6,7].

The high binding affinity of phenylboronic acid (PBA) to sialylated epitopes has been explored for selective targeting of bioactive molecules to tumor cells. The approach relies on the molecular interaction of SA with PBA (diol-boronate complex formation), even in the presence of other saccharides [8,9]. The specificity of this interaction has been exploited for delivering various polymeric and lipid-based nanoparticles to SA-overexpressing cancer cells. The approach has received widespread attention from the standpoint of minimizing undesirable drug exposure to off-target and healthy cells. Tang and coworkers [10] demonstrated high penetration of PBA-decorated carboxymethyl chitosan nanoparticles in SH-SY5Y cells and multicellular (SH-SY5Y and H22 cells) spheroids. This enabled superior internalization of loaded doxorubicin into the cell nuclei and consequent dislodgement of target cells from spheroids. Premature drug release during transport through the tumor interstitium can be avoided by designing micellar formulations capable of dissociating under tumor-relevant conditions, as demonstrated in [11]. Here, PBA was linked with the shell-forming block, whereas the drug was attached to the core-forming counterpart using reduction-sensitive thiol linkage. The micelles exhibited high antitumor efficacy on both murine and human hepatoma xenograft models.

In the present study, we describe the attachment of 3-amino phenylboronic acid (APBA) to d-α-tocopheryl polyethylene glycol succinate (TPGS), a nonionic surfactant composed of polyethylene glycol (PEG, molecular weight—1000 Da) linked to α-tocopherol (vitamin E) via a succinate diester bond. With a low critical micelle concentration (CMC, 0.02% *w*/*w*) and a micelle aggregation number close to 100, TPGS has been used as a safe adjuvant in the development of various colloidal formulations. Earlier studies, including ours, have demonstrated that it forms core-shell micelles which remain stable with temperature and concentration [12,13,14,15]. Apart from biosafety, TPGS possesses interesting biological properties relevant to drug delivery in cancer cells [16]. For instance, the tocopheryl moiety exerts a suppressive effect on drug efflux and MDR1 gene activity. In combination with doxorubicin (DOX), it increased the cleavage of caspase-8 and caspase-9 and upregulated the expression of apoptotic genes in cancer cells [17]. Interestingly, earlier investigations have validated the synergism between anticancer drugs and TPGS in cell lines and animal models [18,19]. As a mitochondriotropic material, TPGS drives the migration of carriers from the cytosol to mitochondria, and the ensuing interactions are disruptive to intracellular ATP production [19]. Intrinsic anticancer activity and spontaneous adsorption of TPGS over hydrophobic surfaces have cued the development of multimodal dosage forms. For example, TPGS stabilized near infrared (NIR) responsive materials have shown superior physiological stability and improved cytotoxic response [20,21].

Our functionalization approach involved attachment of APBA onto the carboxylated terminal of TPGS. After characterizing the colloidal properties of TPGS-APBA, the targeting efficiency of micelles was evaluated in MDA-MB-453 cells. Apoptotic ability of the formulation was studied after loading with paclitaxel (PLX), a poorly soluble anticancer drug [22]. Our results show that functionalized TPGS micelles possess superior translocation ability and induce higher apoptosis in SA-expressing cancer cells.

## 2. Materials and Methods

### 2.1. Materials

TPGS (98% pure, ≥25% α-tocopherol, molecular weight—1513 Da) was kindly gifted by Bioplus (Bengaluru, India). Fluorescein isothiocyanate (FITC), 3-amino phenyl boronic acid (APBA), deuterated dimethylsufoxide (DMSO-d_6_) and Triton X100 were purchased from Sigma Aldrich (St. Louis, MO, USA). Pyridine (Py), *N*,*N*-dimethyl formamide (DMF), 4-dimethylaminopyridine (DMAP), succinic anhydride (SA), 1-ethyl-3-(3-dimethylaminopropyl) carbodiimide (EDC) and N-hydroxysuccinimide (NHS) were purchased from S.D. Fine Chemicals (Mumbai), India. PLX was received as a gift sample from Neon Pharmaceuticals (Mumbai, India). Acetonitrile and 1,4-dioxane were purchased from Rankem Laboratories (Mumbai, India). Dulbecco modified Eagle medium (DMEM), fetal bovine serum (FBS) and L-glutamine were purchased from Hi Media Ltd. (Mumbai, India). D_2_O was purchased from Tokyo Chemical Industries (Tokyo, Japan). Dialysis membrane (MWCO < 2000 Da) was procured from Sigma Aldrich (St. Louis, MO, USA). MDA-MB-453 cells were purchased from National Centre for Cell Science (Pune, India). All chemicals and reagents were of analytical grade and used as received.

### 2.2. Methods

**Scheme 1:**
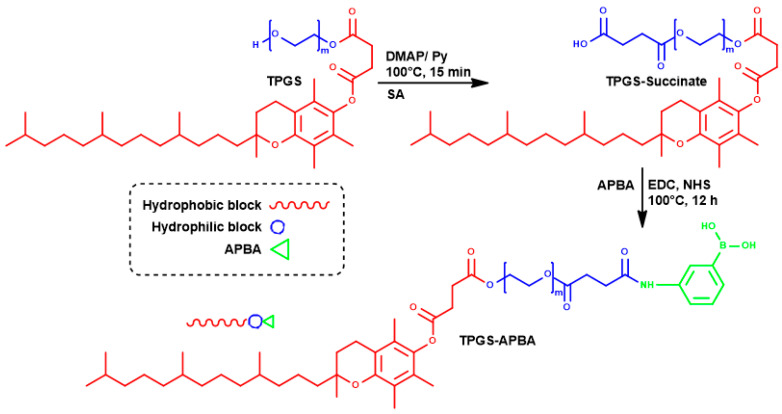
Attachment of APBA at the hydrophilic terminal of TPGS.

Covalent attachment of APBA was performed in two steps: (a) succinylation to introduce a terminal carboxylic acid group in TPGS and (b) attachment of APBA via an amide bond (Scheme 1). TPGS (5.74 g, 10 mmol) was dissolved in DMF and allowed to react with succinic anhydride (1.0 g, 10 mmol) in the presence of DMAP (305 mg, 2.5 mmol), pyridine (2.15 mL, 40 mmol). This was followed by irradiation with microwave at 100 °C (300 W, 15 min) and instant precipitation of the product in iso-propanol (IPA-water, 2:1 *v*/*v*). The product was washed with IPA and dried under vacuum. In the next step, TPGS-succinate (10 mM) was dissolved in DMF and allowed to react with APBA (1.36 g, 10 mmol) at 100 °C for 12 h in the presence of EDC (2.06 g)-NHS (1.0 g) [16]. The reaction mixture was dialyzed against water for 72 h, and TPGS-APBA was finally recovered through lyophilization (Yield 85%).

### 2.3. Characterization

The linkage between TPGS and APBA was characterized through Fourier-transform infrared (FT-IR) and nuclear magnetic resonance (NMR) spectroscopy. The FT-IR spectrum was recorded over the range of 4000–400 cm^−1^ with an average of 32 scans and 4 cm^−1^ resolution (Bruker Alpha, Karlsruhe, Germany). NMR spectra were recorded on a JEOL ECZ600R MHz FT-NMR Spectrometer equipped with ROYAL probe (JEOL, Tokyo, Japan). The sample was dissolved at room temperature in D_2_O (60 mg mL^−1^), and d_3_-acetonitrile was used as internal standard.

Dynamic light scattering (DLS) and small-angle neutron scattering (SANS) were used for the characterization of micelles. DLS data were acquired with a He–Ne laser at a 130° scattering angle (Nano ZS, Malvern Instruments, Malvern, UK). SANS experiments were performed at Dhruva Reactor, Bhabha Atomic Research Centre (Mumbai, India). The angular distribution of neutrons scattered by the sample was recorded using a one meter long, one-dimensional He^3^ position-sensitive detector. For the analyses of structural parameters (supplementary materials), the data were fitted to the spherical particle model [23].

Cellular uptake of micelles was studied using confocal laser scanning microscopy (CLSM, company) and fluorescence-activated cell sorting (FACC) (Malvern Instruments, Malvern, UK). MDA-MB-453 cells were cultured (37 °C, 5% CO_2_) in a DMEM medium supplemented with FBS (10%) and L-glutamine (2 mM). The cells were allowed to attach over a coverslip. The latter were placed into a six-well culture dish containing the culture medium. The medium was replaced with a fresh one (devoid of FBS), and the cells were incubated with FITC-loaded formulations (volume equivalent to 2 × 10^14^ micelles) for 4 h. After this, the cells were washed to remove free micelles and fixed with 4% formaldehyde. Nuclei were stained with 4′,6-diamidino-2-phenylindole (DAPI) in the dark, and images were acquired with a confocal laser microscope (Zeiss 510 META, Jena, Germany). The uptake was quantified through FACS analysis. Here, the cells (5 × 10^4^) were grown over six-well culture plates. After the treatment and washing as discussed above, the cells were suspended in ice-cold calcium/magnesium-free phosphate buffer saline and stored on ice until the analyses. A minimum of 10,000 cells were counted in the gated region for each sample (Malvern Instruments, Malvern, UK). Comparative efficacy of TPGS and TPGS-APBA micelles was evaluated at identical PLX doses (250 ng) using an Annexin V-FITC—propidium iodide (AV/PI) assay [22].

## 3. Results and Discussion

**Figure 1:**
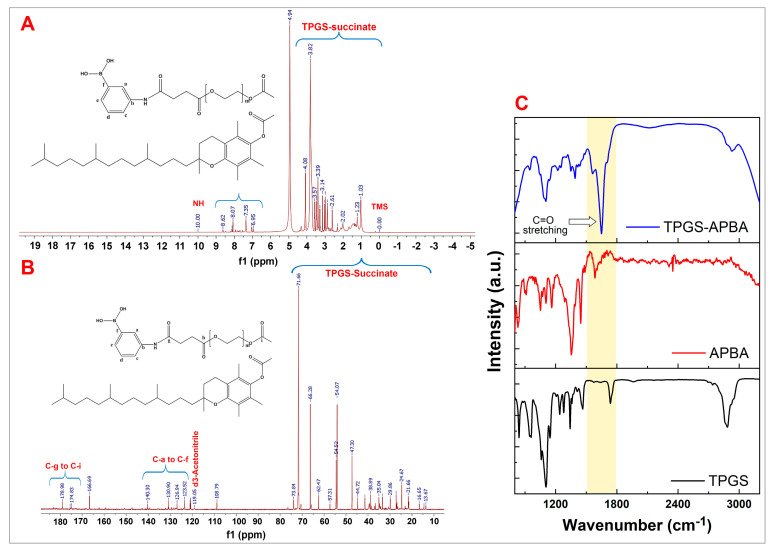
Characterization of functionalized TPGS using proton (**A**) and carbon (**B**) NMR (TMS—chlorotrimethylsilane). The right panel shows FT-IR spectral changes during APBA attachment (**C**).

The spectral characterization of TPGS-APBA conjugate is shown in Figure 1. The signals observed in ^1^H-NMR over ca. 1.00 to 3.00 ppm emerge from protons in the tail region of TPGS. Sharp signals at 3.39 to 3.82 represent methylene (–CH_2_) protons of the succinate linker. Chemical shifts at 4.08 and 4.20 ppm represent –CH_2_ protons of the PEG segment [16,24]. The aromatic region of APBA can be confirmed due to the chemical shifts in the range of 6.95 to 8.62 ppm. The signal observed at 10.00 ppm represents the proton of –NH– of the APBA moiety (Figure 1A). These interpretations are well supported by ^13^C NMR data (Figure 1B). It shows the peaks representing –CH_3_ (13.67, 14.55, 16.65, 21.65, 23.12 and 24.66 ppm), –CH_2_ (26.44 to 76.44 ppm) and –CH (ca. 25.0 ppm) groups of TPGS. The signals at 174.83 to 178.98 ppm represent carbonyl carbons (succinate linker) [25]. Aromatic carbons of APBA are visible in the range of 123.52 to 140.30 ppm [26]. Altogether, the evidence of amide proton (δ 10.00 ppm) and an isolated peak of amide carbon (δ 178.98 ppm) confirm that the linkage between APBA and TPGS successfully occurred [27].

The FT-IR spectrum of TPGS displayed a distinctive band at around 1732 cm^−1^, which asserted the presence of carbonyl (C=O) functional groups of the TPGS molecule, as well as successful conjugation with succinic anhydride. Peaks at 1100 and 2930 cm^−1^, respectively, correspond to C–O–C and –CH_2_ groups in TPGS. Similarly, pure APBA shows N–H (1560 cm^−1^), B–O (1640 cm^−1^) and –OH (≈3300 cm^−1^) stretching vibrations [28]. In the case of TPGS-APBA conjugate, reaction centers representing neat TPGS (C–O–C) and APBA (N–H) disappeared. This was accompanied by a new strong peak close to 1650 cm^−1^ (C=O stretching) [24] pointing towards the appearance of an amide linkage between TPGS and APBA (Figure 1C).

**Figure 2:**
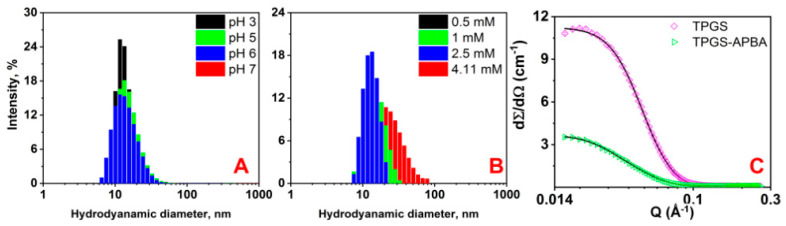
DLS data of TPGS-APBA micelles showing the effect of pH changes (**A**) and PLX encapsulation (**B**). Difference in neutron scattering profiles of TPGS and TPGS-APBA is shown in (**C**). Solid lines correspond to the fits in core-shell model. The concentration was fixed as 2%.

Next, we studied the colloidal properties of TPGS-APBA conjugate in terms of average hydrodynamic diameter (D_h_) and zeta potential. Micelles were prepared in deionized water by direct dissolution of the conjugate under mild orbital stirring. Measurements were performed after equilibrating the samples at least overnight (room temperature). The conjugate quickly self-assembled in water to produce micelles with a unimodal distribution profile (polydispersity index < 0.2). DLS data showed that micelle size did not change in spite of increased molar mass (data not shown). However, there were disagreements on the changes in the size of micelles developed with functionalized amphiphiles. For example, the average size of TPGS-2000 micelles shifted from 30 to 51 nm upon attachment of folic acid [29]. Similarly, Brandt and coworkers [30] recorded a 50% higher size of micelles after functionalization of the block copolymer with folic acid. Moreover, folic acid imparted negative zeta potential to the micelles, which was sustained in the low pH (3–6) region as well. In our case, the zeta potential was found to be −2.14 mV, apparently because of the nonionic nature of the surfactant. This can be the reason why average size remained invariant with changes in pH (Figure 2A).

Micelles showed solubilization of PLX until 2.5 mM without any changes in the average hydrodynamic diameter (D_h_). This can be ascribed to its strongly hydrophobic core, which favors the encapsulation of poorly water soluble molecules [29]. This principle has been explored extensively to tailor the molecular characteristics of amphiphiles. For instance, the loading of PLX improved as the amine terminal of bovine serum albumin was hydrophobized with increasing molar proportion of octyl groups. Here, high loading emanated from the synergism between micellar encapsulation and binding interaction between the drug and the non-polar region [31]. It is argued that hydrophobic interactions are augmented during drug encapsulation, and it sometimes triggers contraction of the core, as reported earlier by our group in the TPGS-carbamazepine system [13]. These differences can be related to the molecular size of payloads: small hydrophobic molecules impose less steric constraints and, hence, offer better engagement with the core block. However, as solubilization reached saturation (4.11 mM), micelle size increased (Figure 2B). The effect of high PLX load on the core and shell regions of the micelle has been described using SANS analysis.

The neutron scattering profile of TPGS micelles points toward the spherical shape of micelles. Except the reduction in intensity, the scattering properties were retained post-functionalization (Figure 2C). The reduction in the scattering intensity corresponds to the number density of micelles [32]. Our results suggest that, despite greater opportunity of hydrogen bonding in the presence of terminal diol groups, the aggregation parameters of TPGS-APBA remained consistent. Consistency of the aggregation number implies that APBA allowed better engagement of head groups with neighboring water molecules without expanding the corona (Table 1).

**Figure 3:**
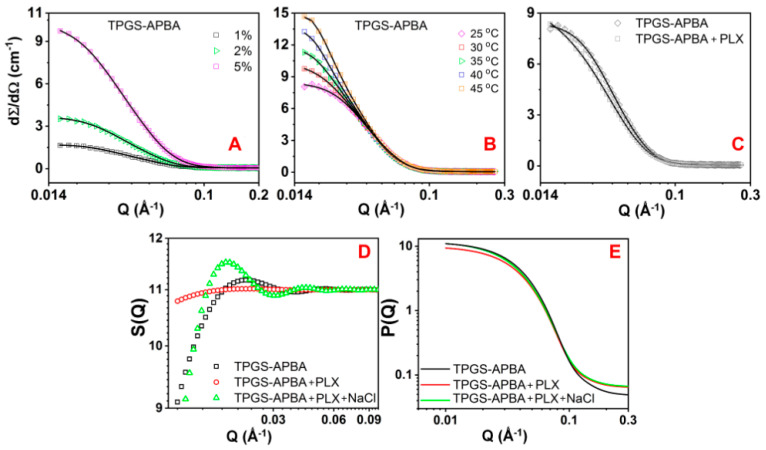
SANS profile showing the effect concentration (**A**), temperature (**B**) and PLX incorporation (**C**) in TPGS-APBA micelles. Bottom panel shows changes in the structure (**D**) and form (**E**) factors of TPGS-APBA micelles upon incorporation of NaCl (0.9%) and PLX.

SANS data further reveal that micelle size was maintained, regardless of conjugate concentration and temperature of the medium. The micelles were stable over the temperature range of 25 °C to 45 °C. The aggregation number remained invariant of concentration and temperature (Figure 3A,B; Table 1). However, considerable changes in the scattering parameters were recorded upon PLX incorporation. The data suggest that PLX incorporation triggered a drop in the volume fraction. It points toward the participation of fewer surfactant molecules in micelle formation. Instead, these might have existed in molecularly dissolved forms. PLX load at saturated level triggered a significant change in the micelle size. A more critical examination suggests about a twofold expansion in corona without any changes in core size (Figure 3C; Table 1). This can be explained by taking into account the aromaticity and large molecular size of PLX. Its aromatic center would have engaged with APBA via π–π stacking. While the insertion of planar molecules (carbamazepine, for instance) may not cause observable expansion, PLX exhibits a different stacking pattern because of its three separate aromatic centers. In addition to face-to-face interactions (π−π), three aromatic rings acquire perpendicular (CH−π forces) and other unconventional orientations simultaneously [33]. We argue that these orientations allow the bulky PLX molecule to intersect through the adjoining head groups and bring about expansion in the corona.

Structural changes in corona were verified by extracting the structure factor *S*(*Q*) and the form factor *P*(*Q*) from the fitted SANS data of micelles [23,34]. In line with the above arguments, PLX-loaded micelles showed substantial deviation in *S*(*Q*), along the higher-length scale (low *Q*) region. This indicates an increase in the size of micelles. It is important to note the partial recovery of *S*(*Q*) upon addition of NaCl (0.9%). This can be ascribed to changes in hydrophilicity of the corona during the salting out effect. Nevertheless, the dynamics at the interface did not bring about any difference in *P*(*Q*); the curves almost coincided with each other (Figure 3D,E). This led us to the conclusion that the shape of micelles was retained even with perturbations in the corona.

**Figure 4:**
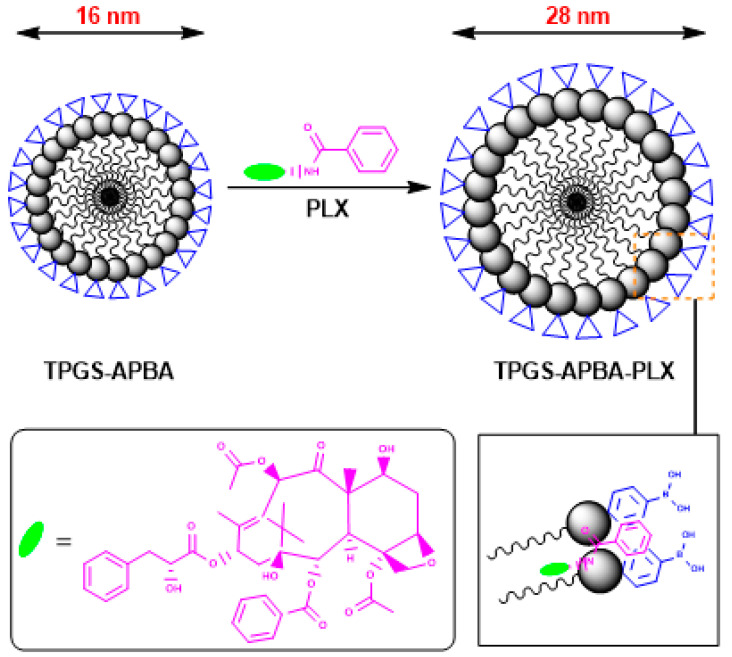
A proposed scheme showing intercalation of PLX molecules in the head group region to stack over APBA via π–π forces.

The participation of PLX is evident from a slight increase in *N_agg_* and a reduction in the number density of micelles. The latter is attributable to the increased micellar dimension explained earlier. An opposite effect has been reported upon functionalization of the hydrophobic block with aromatic substituents. For example, swelling of the micelle core was reported by Nottelet and coworkers [35], following π–π stacking between curcumin (CUR, a polyphenol) and aromatic benzylthioether (BTE) groups. BTE groups were appended to the polycaprolactone (PCL) segment of PEG-(PCL)_8_ star block copolymer. Hydrophobic interactions within the core allowed accommodation of additional CUR molecules and caused an upshift in the size distribution of micelles. Nonetheless, π–π interactions have been implicated in improving micelle stability and controlling the release rate of the encapsulated drug [36]. Hence, we suggest that drug molecules were mobilized to the hydrophobic interior of micelles until saturation. A fraction of the load associated with the head groups in the presence of APBA (Figure 4). A similar association has been reported at the surface between folic acid (targeting ligand) and methotrexate in PEG-PCL micelles [30].

**Figure 5:**
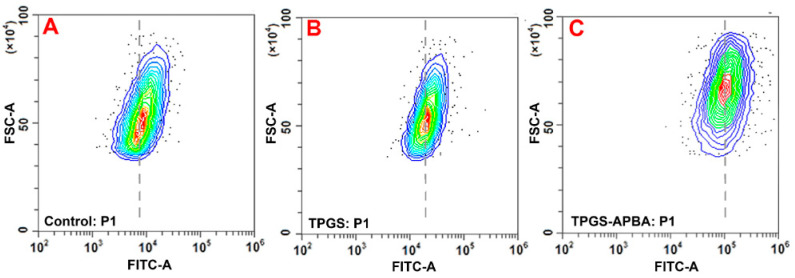
Effect of APBA attachment upon trans-cellular uptake of micelles. Shown are the FACS data for untreated cells (**A**), and those incubated with TPGS (**B**) and TPGS-APBA (**C**) micelles. The cells in (**B**) and (**C**) were incubated with FITC-loaded micelles for 4 h.

The uptake and translocation abilities of functionalized micelles were studied in MDA-MB-453 cells using FACS and confocal microscopy. FACS data show a clear difference between the cellular uptake of TPGS and TPGS-APBA micelles (Figure 5A–C). It elucidates that attachment of APBA correlated with improved affinity of cells for the micelles. About a tenfold increase in the cellular uptake of APBA-functionalized TPGS micelles was recorded. This underlines the role of the proposed diol-boronate interaction in facilitating the transport of carriers. The specificity of the APBA-mediated interaction was validated by pre-incubating the cells with free APBA (5 µM) as a competitive inhibitor, which caused about 62% inhibition in the uptake. Similarly, more than 85% reduction in the uptake of micelles was recorded at 4 °C (data not shown). While binding of free PBA would have rendered the majority of diol sites inaccessible to functionalized micelles in the first case, uptake reduction in the second case strongly suggests that internalization occurred through an energy-dependent, receptor-mediated mechanism [37]. Our results are in agreement with the observations of Tang and coworkers [38] on PBA-functionalized gelatin nanoparticles. The binding ability of nanoparticles to SA-overexpressing cancer cells was reduced upon pre-treatment with free SA.

**Figure 6:**
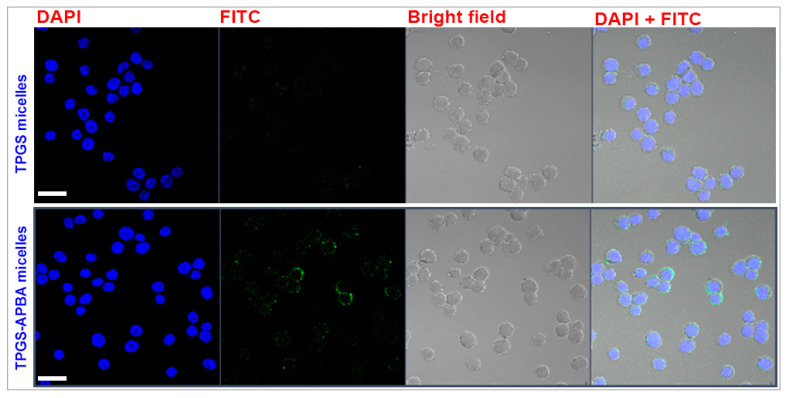
Comparison in the cellular uptake of micelles composed of TPGS (upper panel) and TPGS-APBA (lower panel). Cells were incubated with FITC-loaded micelles for 4 h prior to imaging. (nuclei—blue, micelles—green). The scale bars represent 10 µm.

The difference between the cellular uptake of TPGS and TPGS-APBA micelles can clearly be visualized in confocal microscopic images (Figure 6). Functionalized micelles exhibited comparatively stronger signals (green) of FITC inside the cells. The fact that these signals could not be detected away from cells implies that the micelles were fully internalized without adhering to the membrane. This can partly be attributed to a near-neutral surface charge of the micelles (zeta potential: −2.14 mV), which would have prevented their aggregation at the cell surface. On the contrary, charge-based interaction with extracellular ligands sometimes causes aggregation of carriers at the cell surface. An earlier study demonstrated the accumulation of highly cationized polymeric nanoparticles (zeta potential > 45 mV) at the surface of cells overexpressing SA residues [22].

The signals, however, did not merge with the nuclear stain (blue), implying that micelles did not enter the nucleus. These observations match an earlier report which showed cytosolic accumulation of TPGS micelles functionalized with cysteine-arginine-glycine-aspartic acid-lysine (CRGDK), a cell-penetrating peptide. After de-micellization, the payload initiated apoptosis through de-stabilization of the mitochondrial membrane potential [39]. Some groups have demonstrated the superiority of small-sized unimers (D_h_: 3–7 nm) on account of their nuclear localization ability. For example, a recent study by Zhang et al. [40] showed that PBA-decorated unimers co-localized with the nuclei of HepG2 tumor cells. The unimers were composed of a multi-arm polyphosphoester copolymer appended with PBA end-groups. While copolymers with large and hyperbranched structures offer ample opportunity of drug loading, the same may not be possible for linear amphiphiles like TPGS.

**Figure 7:**
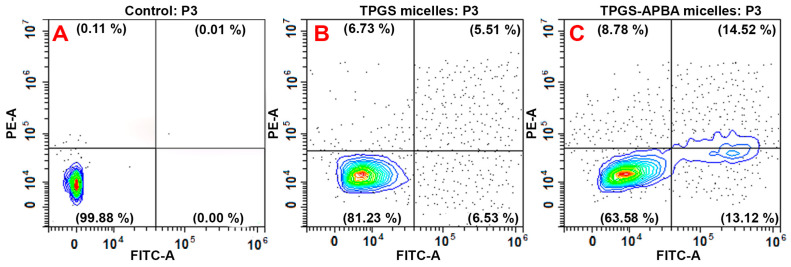
Results of apoptosis assay. Phosphate buffer saline was used as negative control (**A**)**.** The data show a comparison between apoptotic ability of PLX delivered in TPGS (**B**) and TPGS-APBA (**C**) micelles. Top and bottom (right) quadrants show the cells positive for AV (early apoptosis) and AV + PI (dead cells), respectively. Healthy cells remained negative to both FITC and PI.

Drug targeting in cancer aims at focused destruction of a selective group of cells. Hence, we analyzed the consequences of improved cellular uptake of micelles in terms of improvement in the cytotoxic action of PLX. Our analysis is based on Annexin V-FITC and propidium iodide (AV/PI) assay. Cells were incubated with PLX-loaded carriers for 2 h, and apoptosis caused during the treatment is displayed in Figure 7A–C. The percentage of cells leaning towards apoptosis was clearly higher with TPGS-APBA micelles. About a twofold increase in the population of apoptotic cells can be noticed. It is important to note that direct drug exposure to intra-cellular organelles is prevented by entrapment of nanoparticulate carriers in endosomal vesicles and subsequent digestion in lysosomes [41]. Looking at the apoptosis data, it is possible that the endosomes were overwhelmed by rapid entry of micelles in high number density. After entering the cytosol, drug-loaded micelles interacted with intracellular targets to stimulate apoptosis. Another possible mechanism relies on disassembly of micelles, which allowed the diffusion of PLX into the nucleus and cytosolic organelles. In this case, drug diffusion would have been driven by the concentration gradient leading to quick intracellular accumulation of micelles. Collectively, our observations elucidate that the death rate of cancer cells can indeed be improved with the use of APBA-functionalized carriers. It will be interesting to investigate the changes in affinity interaction of micelles at varying proportions of TPGS and TPGS-APBA.

## 4. Conclusions

We demonstrate successful synthesis, self-assembly and application of TPGS-APBA conjugate for active targeting to cancer cells. The construct self-assembled in water to form nanoscale spherical core-shell micelles exhibiting narrow size distribution. The micelles remained stable against temperature, pH and salt exposure within physiological range. Confocal microscopy and FACS studies revealed that the presence of PBA groups in the head group region enabled high translocation of micelles into cancer cells in vitro. This was accompanied by a stronger apoptotic response of PLX-loaded micelles. Interestingly, the loading of PLX was partly facilitated through π–π interaction with PBA groups in the head group region of micelles. Collectively, our results suggest that PBA-based actively targeted carriers can be employed to deliver a high drug payload to cancer cells. This platform can be used to detect and eliminate cancer cells in the pre-invasive state.

## Figures and Tables

**Table 1:** Structural parameters of TPGS-APBA micelles obtained after fitting the data into spherical core-shell model.

Sample		*R_c_*	*R_hs_*	Φ	PD	*N_agg_*	*N* (×10^17^)
TPGS (2%)		4.2	6.7	0.044	0.19	121	0.354
TPGS-APBA	2%	4.3	6.5	0.012	0.24	124	0.100
	5%	4.3	6.6	0.021	0.23	122	0.169
	PLX *	4.3	8.4	0.003	0.27	124	0.013
	PLX * + 0.9% NaCl	4.4	12.5	0.063	0.29	129	0.077

*R_c_*—core radius (nm), *R_hs_*—hard sphere radius (nm), Φ—volume fraction, *N_agg_*—aggregation number, *N*—number density of micelles (cm^−3^). * micelles were allowed to equilibrate with the drug for 48 h.

## Supplementary Materials

The following are available online at https://www.mdpi.com/2073-4360/12/10/2258/s1.
