# Supplementary material for: Synthesis, Colloidal Characterization and Targetability of Phenylboronic Acid Functionalized α-Tocopheryl Polyethylene Glycol Succinate in Cancer Cells
Source: Polymers (Basel). 2020 Oct 1;12(10):2258. doi: 10.3390/polym12102258 (PMC7600591; doi:10.3390/polym12102258)
Supplement: Supplementary file 1 [file polymers-12-02258-s001.pdf]

polymers

MDPI

Article

Supplementary Material:

Synthesis, Colloidal Characterization and Targetability of Phenylboronic Acid Functionalized α-Tocopheryl Polyethylene Glycol Succinate in Cancer Cells

Sanjay Tiwari [1], Jayant Sarolia [1], Vrushti Kansara [1], Nishith A Chudasama [2], Kamalesh Prasad [2], Debes Ray [3], Vinod K Aswal [3] and Pratap Bahadur [4,*]

[1] Maliba Pharmacy College, Gopal-Vidyanagar Campus, Uka Tarsadia University, Surat 394350, India; sanjay.tiwari@utu.ac.in (S.T.); jayantsarolia@gmail.com (J.S.); v.kansara12@gmail.com (V.K.)
[2] Natural Products & Green Chemistry Division, Central Salt and Marine Chemicals Research Institute, Bhavnagar 364002, India; chudasamnishith@gmail.com (N.A.C.); kamlesh@csmcri.res.in (K.P.)
[3] Solid State Physics Division, Bhabha Atomic Research Centre, Mumbai 400085, India; debes@barc.gov.in (D.R.); vkaswal@barc.gov.in (V.K.A.)
[4] Department of Chemistry, Veer Narmad South Gujarat University, Surat 395007, India
* Correspondence: pbahadur2002@yahoo.com

Received: 17 September 2020; Accepted: 27 September 2020; Published: 1 October 2020

Analysis of SANS data:

The differential scattering cross-section per unit volume ($d\Sigma/d\Omega$) as measured for a system of monodisperse particles in a medium can be expressed as [1-3]

$$\left(\frac{d\Sigma}{d\Omega}\right)(Q) = nV^2\left(\rho_p - \rho_s\right)^2 P(Q)S(Q) + B \tag{1}$$

where n denotes the number density of particles, ρ_p and ρ_s are, respectively, the scattering length densities of particle and solvent and V is the volume of the particle. $P(Q)$ is the intraparticle structure factor and $S(Q)$ is the interparticle structure factor. B is a constant term representing incoherent background, which is mainly due to the hydrogen present in the sample.

Intraparticle structure factor $P(Q)$ is decided by the shape and size of the particle and is the square of single-particle form factor $F(Q)$ as determined by

$$P(Q) = \langle|F(Q)|^2\rangle \tag{2}$$

For a spherical particle of radius R, $F(Q)$ is given by

$$F(Q) = 3\left[\frac{\sin(QR) - QR\cos(QR)}{(QR)^3}\right] \tag{3}$$

$S(Q)$ describes the interaction between the particles present in the system and it is the Fourier transform of the radial distribution function $g(r)$. $g(r)$ gives the probability of finding the center of another particle at a distance r from the center of a reference particle. $S(Q)$ has been calculated using the mean spherical approximation developed by Hayter and Penfold [4]. In this approximation, the particle (in this case, micelle) is treated as a rigid equivalent sphere of diameter d interacting with another micelle through a screened coulomb potential $u(r)$ given by the relation

$$u(r) = u_0 d \exp\left[-\kappa(r-d)\right]/r,\ r > d \tag{4}$$

Where u_0 is the potential at $r = d$ and the Debye-Huckel inverse screening length κ is evaluated by using the expression

$$\kappa = \left(\frac{8\pi N_A e^2 I}{10^3 \varepsilon k_B T}\right)^{1/2} \tag{5}$$

Where N_A, e, I, ε, k_B and T denote Avogadro number, electronic charge, ionic strength of the micellar solution, dielectric constant of the solvent, Boltzmann constant and absolute temperature, respectively.

The polydispersity in size distribution of particle is incorporated using the following integration [5]

$$\frac{d\Sigma}{d\Omega}(Q) = \int \frac{d\Sigma}{d\Omega}(Q,R) f(R) dR + B \tag{6}$$

where $f(R)$ is the size distribution of the vesicles and usually accounted by a log-normal distribution as given by

$$f(R) = \frac{1}{\sqrt{2\pi}R\sigma} \exp\left[-\frac{1}{2\sigma^2}\left(\ln\frac{R}{R_{med}}\right)^2\right] \tag{7}$$

where R_{med} is the median value and σ is the standard deviation (polydispersity) of the distribution. The mean radius (R_m) is given by

$$R_m = R_{med} \exp(\sigma^2/2). \tag{8}$$

The data have been analyzed by comparing the scattering from different models to the experimental data. Throughout the data analysis, corrections were made for instrumental smearing, where the calculated scattering profiles smeared by the appropriate resolution function to compare with the measured data [6]. The fitted parameters in the analysis were optimized using nonlinear least-square fitting program to the model scattering [7].

Calculation of aggregation number and number density of micelles:

The aggregation number (N_{agg}) of micelles defines the total number of surfactant molecules forming pure or mixed micelles. N_{agg} from SANS measurements has been calculated by using the following relationship:

$$N_{agg} = V_m/V_h \tag{9}$$

where V_m is the micellar volume and is given by

$$V_m = 4\pi R_c^3/3 \tag{10}$$

R_c is the core radius of micelles. V_h is the molecular volume of hydrophobic tail of the TPGS. In case of mixed micelles, it represents the hydrophobic part of the mixture and is given by:

$$V_h = V_h^1 + C_2/C_1 V_h^2 \tag{11}$$

where V_h^1 and V_h^2 are the molecular volumes of hydrophobic part of the TPGS and ILs component respectively. C_1 and C_2 are the concentrations of TPGS and ILs component respectively.

Number density micelles calculation

$$\Phi = NV \tag{12}$$

$$\Phi = \text{Volume fraction of micelles} \tag{13}$$

$$V = \text{micelle volume } (4\pi R^3/3\ ;\ R = \text{micelle radius}) \tag{14}$$

$$N = \text{Micelles number density} \tag{15}$$

References:

1. Hayter, J.B. and Penfold J. Determination of micelle structure and charge by neutron small-angle scattering. *Colloid Polym. Sci.* **1983,** *261,* 1022.
2. Kaler E W. Small-angle scattering from colloidal dispersions. *J. Appl. Cryst.* **1988,** *21,* 729.
3. Ray D, Aswal V K, Kohlbrecher J. Synthesis and characterization of high concentration block copolymer-mediated gold nanoparticles. *Langmuir* **2011,** *27,* 4048.
4. Hayter J B, Penfold J. An analytic structure factor for macroion solutions. *Mol. Phys.* **1981,** *42,* 109.
5. Pedersen J S. Analysis of small-angle scattering data from colloids and polymer solutions: modeling and least-squares fitting. *Adv. Colloid Interface Sci.* **1977,** *70,* 171.
6. Pedersen J S, Riekel C. Resolution function and flux at the sample for small-angle X-ray scattering calculated in position–angle–wavelength space. *J. Appl. Cryst.* **1991,** *24,* 893.
7. Bevington P R, Robinson D K. Data reduction and error analysis for the physical sciences McGraw-Hill. *New York,* **1969,** *19692,* 235-242.

© 2020 by the authors. Submitted for possible open access publication under the terms and conditions of the Creative Commons Attribution (CC BY) license (http://creativecommons.org/licenses/by/4.0/).
